# Male Victims at a Dutch Sexual Assault Center: A Comparison to Female Victims inCharacteristics and Service Use

**DOI:** 10.1177/08862605211015220

**Published:** 2021-05-13

**Authors:** Milou L. V. Covers, Janna Teeuwen, Iva A. E. Bicanic

**Affiliations:** 1 National Psychotrauma Center for Children and Youth, University Medical Center Utrecht, Lundlaan 6, 3584 EA Utrecht, The Netherlands

**Keywords:** male victims, sexual assault center, rape, service use

## Abstract

Recently, there has been an increase in referrals of male victims of sexual assault to interdisciplinary sexual assault centers (SACs). Still, there is limited research on the characteristics of men who refer or are referred to SACs and the services they need. To facilitate the medical, forensic, and psychological treatment in SACs, a better understanding of male victims is indispensable.

The first aim of the study was to analyze the victim and assault characteristics of male victims at a Dutch SAC, and to compare them to those of female victims. The second aim was to analyze and compare SAC service use between male and female victims.

The victim characteristics, assault characteristics, and service use of 34 male victims and 633 female victims were collected in a Dutch SAC. *T*-tests and chi-square tests were used to analyze differences between male and female victims.

No differences between males and females in victim or assault characteristics were found. Most victims received medical and psychological care, with no differences between male and female victims. Female victims were more likely to have contact with the police, but no differences in reporting or forensic medical examinations between males and females were found.

These findings indicate that SACs can and do provide equal services to male and female victims, and that the current services are suitable for male victims as well. However, a focus on educating and advising male victims about police involvement is advisable.

## Introduction

In the last 20 years, hospitals and mental health organizations across Europe have established specialized sexual assault centers (SACs) (Bicanic et al., 2014; Kerr et al., 2003; Schei et al., 2003; Vandenberghe et al., 2018). These centers provide acute medical, forensic, and psychological care to anyone who believes that they have been the victim of sexual assault. Victims of sexual assault (i. e., oral, anal, or vaginal penetration without consent, unwanted sexual touching or kissing, hands-off, or online sexual abuse) can refer themselves to these centers in the first week after the assault (Centrum Seksueel Geweld, 2020). Alternatively, medical and psychological professionals or police officers can refer someone to the SACs. Since the start-up of these SACs, the victims who use the services in these centers have been mainly female,and studies have aimed to identify the needs of these female victims in order to provide suitable care (Bicanic et al., 2014; Kerr et al., 2003; Schei et al., 2003). However, yearly, 4–7%of men in the Netherlands suffer sexual assault as well (de Graaf & Wijsen, 2017). The underrepresentation of male victims at SACs has resulted in limited research and knowledge on the medical, forensic, or psychological needs of men who seek help in the immediate aftermath of sexual assault.

In this study, we examined the characteristics of male victims and their assault in sexual assault victims who refer to a Dutch SAC, as well as the SAC services that they use. Moreover, we explored differences between male and female victims in victims characteristics, assault characteristics, and service use.

### Male Victims’ Disclosure

Research has found the main reason for the underrepresentation of male victims at SACs to bethe delay or absence of disclosure:Male victims are less likely to disclose their assault and seek help than female victims (de Graaf & Wijsen, 2017; Monk-Turner & Light, 2010). This barrier to disclose is related to commonly held myths surrounding male sexual assault victimization (Easton et al., 2014; Lowe & Rogers, 2017; Sorsoli et al., 2008; Turchik & Edwards, 2012). These rape myths include that men cannot be victims, because victims are considered as weak and society expects men to be strong (Dorahy & Clearwater, 2012; O’Leary & Barber, 2008; Young et al., 2016). Other myths imply that when men become victim of sexual violence, they do not develop any distress and may even find it pleasurable (Peterson et al., 2011). These rape myths attribute responsibility for the assault to male victims. In fact, a vignette study found that male victims were more likely to be blamed for their assault than female victims (Davies et al., 2009). Male victims also blame themselves for the assault andtherefore experience feelings of shame (Dorahy & Clearwater, 2012). Moreover, the rape myths cause male victims to believe they should have experienced pleasure from the assault, resulting in long term crises with sexual orientation and masculinity in more than half of all male victims (Walker et al., 2005). Crucially, these male rape myths impact male victims’ perception of available help: There is evidence to suggest that male victims do not seek help because they expect professionals to disbelieve or blame them (Depraetere et al., 2018). Thus, male rape myths influence male victims’ disclosure and preclude immediate care after sexual assault.

### Male Victims at SACs

Nonetheless, the Dutch SACs have seen an increase in male referrals over the last few years, increasing from 8% of all referrals in 2017 to 12% in 2019 (Centrum Seksueel Geweld, 2018; Centrum Seksueel Geweld, 2020). This development is important, as research shows that victims who receive coordinated care within a week after the assault are more likely to obtain the resources needed to facilitate their recovery (Campbell et al., 2001). However, this increase in male referrals raises the question whether the SACs’typically female-focused services align with the service needs of male victims. To answer this question,it is important to look at research on male victims’ service use and how this compares to female victims’.

A limited body of research reports on the male victims’ use of SAC services. Research at Canadian (Du Mont et al., 2013), Danish (Larsen & Hilden, 2016), and American SACs (Kimerling et al., 2002; Riggs et al., 2000) has consistently found the need for treatment of genital or rectal injuries in one third of male victims. About half of male victims seeking help at SACs receive forensic medical examination (FME) to collect evidence to potentially use in court (Du Mont et al., 2013; McLean et al., 2005) and about half of male victims at SACs report the assault to the police (Kimerling et al., 2002; Larsen & Hilden, 2016). The use of psychological care by men has rarely been studied and findings are mixed: A SAC in Canada reported that 76% of men sought counselling (Du Mont et al., 2013; *n*=38), in contrast to 48% in the United Kingdom (McLean et al., 2005; *n* = 376). Although this difference may be explained by the difference in sample size, these contrasting findings underline the importance of further research.

When comparing the characteristics of male victims’ assault to those of female victims, three studies report on these differences within SACs. First, Riggs et al. (2000) and McLean et al. (2005) found that men who refer to SACs were more likely to have multiple assailants than women. Mclean et al. (2005) also reported that men were more likely to be assaulted in public places, but found no difference in the use of force, violence, or weapons between male and female victims. In contrast to this, Kimerling et al. (2002) and Larsen and Hilden (2016) found that female victims had more often suffered injuries than male victims. Still, men were less likely to report their assault to the police (Kimerling et al., 2002; McLean et al., 2005). Lastly, more male than female victims suffered from pre-existing psychiatric disorders (Kimerling et al., 2002).

It should be noted that across these studies on male victims of sexual assault, male victims under the age of 12 are often referred to as victims of child sexual abuse and therefore left out of analysis (e. g.,DuMont et al., 2013; Masho & Alvanzo, 2010; McLean et al., 2005). However, in the Netherlands, victims of all ages are welcomed by the SACs and receive equal care.

### This Research

To facilitate the medical, forensic, and psychological treatment of men in SACs, a better understanding of these victims, their assaults and service use is needed. The present study aims to examine the victims and assault characteristics and service use of male victims who refer to or are referred to a Dutch SAC, and to compare them to those of female victims. First, the victims characteristics, consisting of age and frequency of pre-existing mental health care, and assaultcharacteristics, consisting of type of assault, frequency of physical injury, physical violence, verbal violence, multiple assailants, and assaults in public places, of the male victims will be analyzed and compared to those of female victims. Second, the use of SAC services, including medical services, forensic services, crisis counselling, and referrals to mental health services of male victims will be analyzed and compared to female victims.

## Method

### Participants

The present study was conducted at a Dutch SAC. The Dutch SACs are interdisciplinary centers combining 24×7 acute medical, forensic, and psychological services for anyone who believes that he or she has been a victim of sexual assault within the last 7days. The participants of this study either presented themselves at one of the sixteen Dutch SACs (location redacted for peer review) or were referred by the police, medical practitioners, mental health professionals, or people from their own network.

The medical services of the SAC entail treatment for physical injuries, pregnancy testing, and the testing, prevention, and treatment for sexual transmitted diseases (STD). The forensic services of the SAC exist of collecting evidence through FME for victims who wish to report their assault to the police. The SACs work closely together withdetectives from the specialized sexual assault department of the police. The psychological services of the SAC entail a psychological stress reaction monitoring process during the first four weeks post-assault. This “watchful waiting” approach is recommended as early intervention after a traumatic event (National Institute for Clinical Excellence, 2005). The watchful waiting protocol is carried out by a trained case manager via phone. When the case manager detects a need for further diagnostics and/or treatment, the victim is referred to mental health services for trauma-based treatment.

### Procedure

At admission to the SAC, information concerning victim characteristics, assault characteristics, and the use of services were registered into the victims’ medical files (all medical information such as injury and medication use) and SAC patient files (victim and assault characteristics, and SAC service use) by the case managers. There was no standardized method for collecting this information, but case managers registered all information that the victim provided, with the victim’s verbal consent. For the present study, a trained case manager coded all available information into a database. Only the case files of victims who referred to the SAC at [one location of the SACs, redacted for blinded peer review] were available for analysis. A total of 705 victims, including 44 men and 661 women, were seen at this SAC between January 2012 and December 2019. All information was anonymized and specific details of the victim and the assault were omitted. According to the Ethical Medical Committee of [redacted for blinded peer review], the Declaration of Helsinki and the Dutch Medical Research involving Human Subjects Act are not applicable to the present study since it uses anonymized patient files.

### Measures

**Victim characteristics.** The present study used the following victim characteristics from the database: gender (male/female), age (continuous), and self-reported current use of mental health services (yes/no). The information about the victim’s gender is not based on biological sex but on the gender identity reported by the victims themselves. In this study gender is described as binary rather than spectral considering every victim identified his or herself as either male or female.

**Assault characteristics.** The victims’ description of the sexual assault was categorized as either unwanted sexual touching (including unwanted kissing) or rape (defined as oral, vaginal, or anal penetration with any body part or object without consent). Physicalviolence during the assault (yes/no), verbal violence during the assault (yes/no), the presence of physical injury (any injuries found during physical examination, including small cuts, bruises, and abrasions; yes/no), multiple assailants (yes/no), and the location of the assault (public/private) were reported as well.

**Service use.** Information was retrieved about the victim’s post-assault use of the SAC services. These variables were coded into yes/no. These variables included the use of any medical services, forensic services, and psychological counselling. Within forensic services, information on contact with the police, FMEs and police reporting were included. Referral to mental health services was included as well.

### Data Analyses

All analyses were specified prior to data collection. Out of 705 victims, 11 were excluded from analyses because the time since the assault was unknown (7men and 4women) and 27 were excluded because there had been no contact between these victims and any of the SAC professionals (3men and 24 women). The remaining dataset consisted of 34 men and 633 women. It should be noted that the age of eight women was unknown, although it was confirmed that they were all adults. These women were not excluded from analysis. The victim characteristics, assault characteristics and service use of male and female victims were reported in frequencies, and chi-square analyses were used to compare these variables between groups (male or female). Where an expected frequency in the chi-square distribution was lower than 5, the Fisher’s exact test was reported. The mean age of male and female victims was compared using an independent sample *t*-test. All analyses were conducted using IBM SPSS version 25. 0.

## Results

The victim characteristics, assault characteristics, and service use are shown in Table 1. There was no difference in age between male and female victims (*t* = –0. 95, *df* = 657, *p* = . 344). Almost half of the male and female victims werereceiving mental health care before the assault. Regarding assault characteristics, most male (87%) victims had experienced rape. The percentage of physical violence in male victims was 27% and 15% had injuries. Fifteen percent of male victims experienced verbal violence. Furthermore, 17% of male assaults involved multiple assailants, and 64% of the male victims were assaulted in a private location. The results of the analyses show no significant differences in any of the victim or assault characteristics between male and female victims. It should be noted that the odds ratios for these characteristics were over 1. 0, indicating that female victims were at higher odds for having pre-existing mental health care and having experienced rape, physical violence, injury, verbal violence, multiple assailants, and assault in a private location. However, the 95% confidence intervals were large which demonstrates little precision in the estimation of the odds ratios.

**Table 1.** Victim characteristics, assault characteristics, and service use of male and female victims of sexual assault

	Men	Women	Chi	Fisher	OR (95% CI)
**Victim characteristics**					
Age	20.88 (10.44)	22.55 (10.01)			
Pre-existing MHC			1.12		1.48 (0.71, 3.09)
Yes	13	270			
No	18	252			
**Assault characteristics**					
Type of assault			.05	1.00	
Touching	4	86			
Rape	26	496			
Physical violence			2.66		2.06 (0.85, 5.00)
Yes	7	202			
No	19	266			
Injury			0.80		1.63 (0.55, 4.82)
Yes	4	135			
No	22	455			
Verbal violence			0.02	1.00	
Yes	4	71			
No	22	362			
Multiple assailants			0.11		1.18 (0.44, 3.16)
Yes	5	113			
No	25	477			
Location			0.98		1.49 (0.67, 3.31)
Public	10	139			
Private	18	373			
**Service use**					
Medical (any)			2.29		1.74 (0.84, 3.60)
Yes	22	482			
No	12	151			
Contact with police			6.45*		2.47 (1.20, 5.07)
Yes	21	499			
No	13	125			
FME			0.78		1.51 (0.61, 3.75)
Yes	8	246			
No	12	245			
Police report			0.65		0.68 (0.27, 1.74)
Yes	13	247			
No	7	195			
Psychological (any)			0.11		0.85 (0.32, 2.24)
Yes	29	525			
No	5	107			
Referral to MHC			0.56		1.31 (0.64, 2.67)
Yes	19	368			
No	14	207			

*Note*. Data is given in mean (sd) or n. * p < .05 ** p < .01. MHC is mental health care. FME is Forensic Medical Examination.

Furthermore, 65% of male victims received medical care and 85% received psychological care at the SAC. Also, 58% of male victims were referred for post-SAC mental health care. The results show no significant difference in the use of medical care, psychological care, or referral between male and female victims. In contrast,a smaller percentage of male victims (62%) than female victims (80%) had contact with the police and this difference was found to be significant. Female victims were two and a half times more likely to have contact with the police at the SAC than male victims, but once the police was involved, there were no significant differences between males and females in FMEs (40% of male victims and 50% of female victims) and police reporting (65% of male victims and 56% of female victims). Within service use, the odds ratios indicate that male victims were more likely than female victims to report to the police and receive psychological care, whereas female victims were more likely to receive medical care and be referred to mental health care. Again, the odds ratios of these services have large confidence intervals.

## Discussion

This study examined and compared the victim and assault characteristics and service use of male and female victims who refer to or are referred to a Dutch SAC. The first aim was to examine the type of assault, frequency of physical injury, physical violence, verbal violence, multiple assailants, assaults in public places, and the age and current use of mental health services of the male victims and to compare them to female victims. The present study found no differences in these victim and assault characteristics between male and female victims. Most victims had experienced rape. Whereas previous research found that one in three male victims who referred to SACs had suffered injury that required treatment (Du Mont et al., 2013; Kimerling et al., 2002; Larsen & Hilden, 2016; Riggs et al., 2000), our study found injuries in only one in seven male victims. This discrepancy may be caused by the fact that in the Netherlands, full body examinations to check for injuries are only standardized for minors, whereas adults must disclose any injuries themselves, while other countries have standardized full body examinations for all ages. When comparing the incidence of physical or verbal violence or injuries between male and female victims, the present study found no differences. This finding is in line with those of McLean et al. (2005), but not with Kimerling et al. (2002). Unlike these studies, our study found no difference between male and female victims in the number of assailants and the location of the assault, nor the use of mental health services. This lack of differences between male and female victims may indicate that male victims with all types of negative sexual experiences refer to the Dutch SAC, and not only those who have experienced extremely violent assaults.

The second aim of the present study was to report the use of SAC services, including medical care, police contact, FME, police reporting, psychological counselling, and referral to mental health care of male victims and to compare them to female victims. This study found that 85% of male victims made use of psychological counselling at the SAC. Previous studies had found varying results, which may be related to the time since the assault. For instance, the study of Du Mont et al. (2013) included victims who referred to the SAC within three days and found a similar percentage as the present study. In contrast, McLean et al. (2005) included all victims, regardless of the time since the assault, and found a lower percentage (48%). This indicates that male victims may be more receptive of psychological care immediately after the assault. In this case, emergency care poses a unique ability to provide psychoeducation and further psychological care that is not present at a later time. Medical care was provided for most victims at admission to the SAC with no differences between male and female victims. The SAC also provides follow-ups for STD screening at 3–4 weeks and 3–6 months, but information about these screenings was not available for the present study. Further research should study the attendance rates of these follow-ups as well, as well as possible risk factors for not attending these follow-ups, including gender.

The current findings support the absence of differences in the use of SAC care between male and female victims, which may indicate either none or equal biases from professionalsacross genders. Nevertheless, there was a difference in overall police involvement, where male victims were less likely to get in contact with the police than female victims. This difference may be caused by commonly held stigmas about masculinity that have been discussed earlier, and the subsequent blame and shame that male victims experience (Davies & Rogers, 2006; Davies et al., 2009; Dorahy & Clearwater, 2012). Male victims may refuse police involvement as they expect that they will be disbelieved, ridiculed, or blamed for their assault (Depraetere et al., 2018; Walker et al., 2005). In another way, the difference in overall police involvement may also reflect differences in the agency that is first consulted by victims after sexual assault. Some victims first consult the police, and others first consult a medical (at the GP’s office or SAC’s emergency room) or psychological professional. Possibly, women are more likely to disclose the assault to the police at first, whereas men more often tell the SACs or their general practitioner about the assault first. Further research is needed to delineate possible differences between male and female victims in the route to SACs. Still, once referred to the SACs, the professionals can influence a person’s choice for service use. Therefore, our findings suggest that for male victims who have not yet contacted the police when referring to SACs, the medical and psychological professionals of the SACs should pay special attention to discussing police involvement with the victims.

For the victims who did have contact with the police, the findings of the present study on FMEs and police reporting were in line with previous research, with about half of male victims receiving a FME and reporting to the police (Du Mont et al., 2013; McLean et al., 2005; Kimerling et al., 2002; Larsen & Hilden, 2016). In contrast to Kimerling et al. (2002), we found no differences between male and female victims, which may reflect fewer stigmas held by the Dutch police about male victims of sexual assault than by American police. This difference may be caused by the specialized training of the detectives who handle sexual assault cases in the Netherlands. Our findings suggest than while male victims may fear or expect not to be taken seriously by the police, the Dutch police provides equal care to both female and male victims of sexual assault. However, further research is needed to explore the stigmas and rape myth acceptance of the Dutch police. Additionally, Dutch police policy for reporting sexual assault stipulates that victims must be fully informed about the process and consequences of reporting sexual assault, in order to facilitate an informed decision for reporting. After this “informed conversation” victims may still choose not to make an official report. Possibly, this policy reduces differences between male and female victims in reporting by reducing victims’ fears.

## Limitations

The current study has several limitations. First, while general information of the victim’s use of services in the SACs was used, there were no details available about this service use. Due to its quantitative approach, the current study does not explore the reasons for this use of services, even though there are several reasons to accept or refuse specific care offers. For example, victims may refuse medical care because of a previous visit to the GP or community health service (Centrum Seksueel Geweld, 2020). Additionally, victims may not be referred for post-SAC mental health care because they claim that they do not need it or because they are already in care. Although this study found the service use to be equal for both male and female victims, there may still be differences in the motivations for accepting or refusing care. Future qualitative research should aim to gain insight in the victims’ different types of motivations. Second, this study has no information on follow-up care, including follow-up medical visits for STD testing and follow up psychological counselling. The timing of referral to mental health care is also unknown, but victims can be referred immediately at intake, after a month of counselling or later. Therefore, no conclusions can be drawn on differences in the intensity or duration of service use between male and female victims. Lastly, the current study has been limited to only male and female victims, considering gender as binary. Specific needs have therefore not been examined for victims who do not identify themselves (exclusively) as male or female, such as transgender or non-binary people.

## Conclusion

The present study found thatmale victims in a Dutch SAC were less likely to get in contact with the police than female victims, indicating the need for medical and psychological SAC personnel to further discuss police involvement with male victims. In contrast to previous research, the present study found no differences between male and female victims in assault characteristics and medical and psychological service use, nor in forensic care after the police was contacted. We can therefore conclude that SAC services are just as suitable for male victims as for female victims, and that the collaboration within the SACs can provide specialized medical, forensic, and psychological care that is equally arranged for, and used by, male and female victims.

## Author Biographies

**Milou L.V. Covers**, MSc, is a researcher for the Sexual Assault Centre in Utrecht and PhD candidate at the University Medical Centre Utrecht. Her research focusses on victims of sexual abuse and PTSD treatment and prevention. She is interested in the development of evidence-based psychological care for people who have recently experienced rape and sexual assault.

**Janna Teeuwen**, MSc, is a psychologist and casemanager at the Sexual Assault Centre in Utrecht. On a daily bases she communicates with recent victims of sexual assault and the multidisciplinary partners of the centre. She is interested in engaging with scientific research to examine more about the strengths and points of improvement of the currently offered help.

**Iva A.E. Bicanic**, PhD, is the head of the National Psychotrauma Centre at the University Medical Centre Utrecht and coordinator of the Dutch network of Sexual Assault Centres. She is an expert in the field of sexual violence. As clinical psychologist, she treats children and adolescents who have experienced trauma, primarily sexual trauma.
